# Supplementary material for: Functional and structural characterization of a two-MAb cocktail for delayed treatment of enterovirus D68 infections
Source: Nat Commun. 2021 May 18;12:2904. doi: 10.1038/s41467-021-23199-5 (PMC8131599; doi:10.1038/s41467-021-23199-5)
Supplement: Supplementary file 1 — Supplementary Information [file 41467_2021_23199_MOESM1_ESM.pdf]

Supplementary Information for:

Functional and structural characterization of a two-MAb cocktail for delayed treatment of enterovirus D68 infections

Chao Zhang[1, 2,#], Cong Xu[3, #], Wenlong Dai[2,#], Yifan Wang[3], Zhi Liu[2], Xueyang Zhang[2], Xuesong Wang[2], Haikun Wang[2], Sitang Gong[1,*], Yao Cong [3, 4,*], Zhong Huang [2,*]

[1] Joint Center for Infection and Immunity, Guangzhou Institute of Pediatrics, Department of Gastroenterology, Guangzhou Women and Children's Medical Center, Guangzhou Medical University, Guangzhou, China

[2] CAS Key Laboratory of Molecular Virology & Immunology, Institut Pasteur of Shanghai, Chinese Academy of Sciences, University of Chinese Academy of Sciences, Shanghai, China

[3] State Key Laboratory of Molecular Biology, National Center for Protein Science Shanghai, Shanghai Institute of Biochemistry and Cell Biology, Center for Excellence in Molecular Cell Science, Chinese Academy of Sciences, University of Chinese Academy of Sciences, Shanghai, China

[4] Shanghai Science Research Center, Chinese Academy of Sciences, Shanghai, China;

[#] These authors contributed equally.

* Corresponding authors.

This Supplementary Information PDF includes:

Figures S1 – S9

Tables S1 – S7

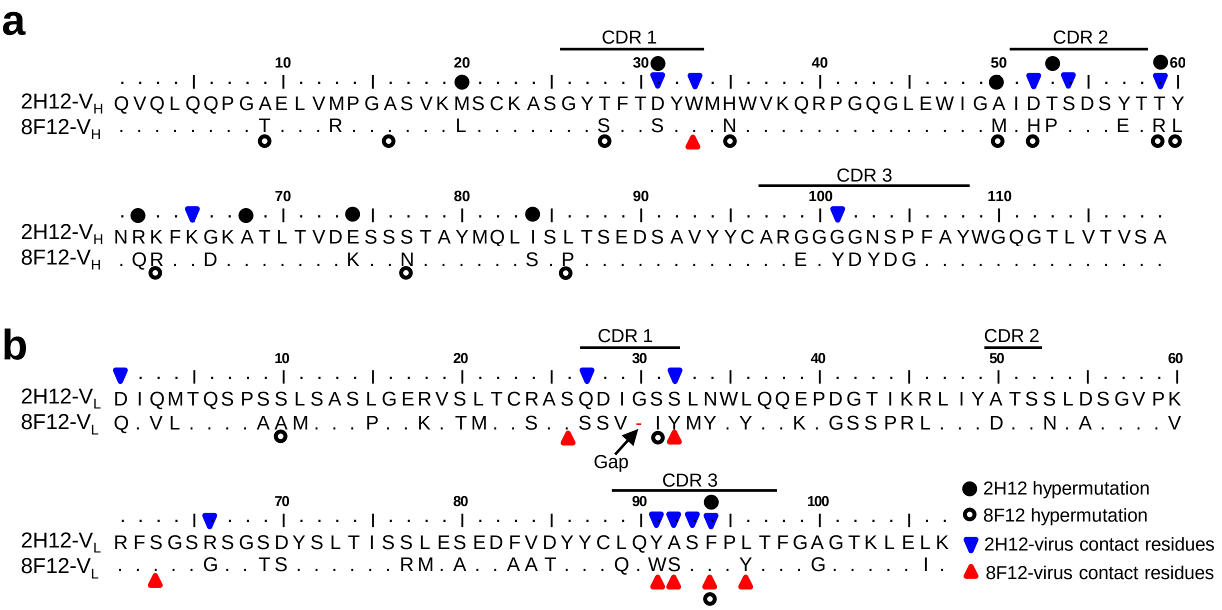

c

MAb	V-gene	D-gene	J-gene	Somatic hypermutation	Identity (%)
2H12-V_H	IGHV1-69*01	IGHD2-1*01	IGHJ3*01	9	92.4
2H12-V_L	IGKV9-120*01	—	IGKJ5*01	1	99.1
8F12-V_H	IGHV1-61*01	IGHD2-4*01	IGHJ3*01	11	90.8
8F12-V_L	IGKV4-55*01	—	IGKJ2*01	3	97.2

Supplementary Figure 1. Analysis of the MAb sequences. Variable domain-encoding sequences of both heavy and light chains of anti-EV-D68 MAbs were determined by 5‘ RACE assay. **(a, b)** Deduced amino acid sequences of heavy-chain variable regions (V_H) **(a)** and light-chain variable regions (V_L) **(b)** of the 2H12 and 8F12 MAbs. Dots represent residues identical to those of variable regions of the MAb 2H12, and red dash is gap. Locations of complementarity determining regions (CDR) were identified using the IgBLAST tool and indicated. Solid and hollow black spheres indicate positions of somatic hypermutation of antibodies 2H12 and 8F12, respectively. The 2H12-EV-D68 and 8F12-EV-D68 contact residues are indicated by blue inverted triangles and red triangles, respectively. **(c)** The closest mouse IgG germline genes were determined using the IgBLAST tool. The number of amino acid changes during antibody somatic hypermutation and the percentage of amino acid identity between our antibody sequences and germline sequences are also shown in this table.

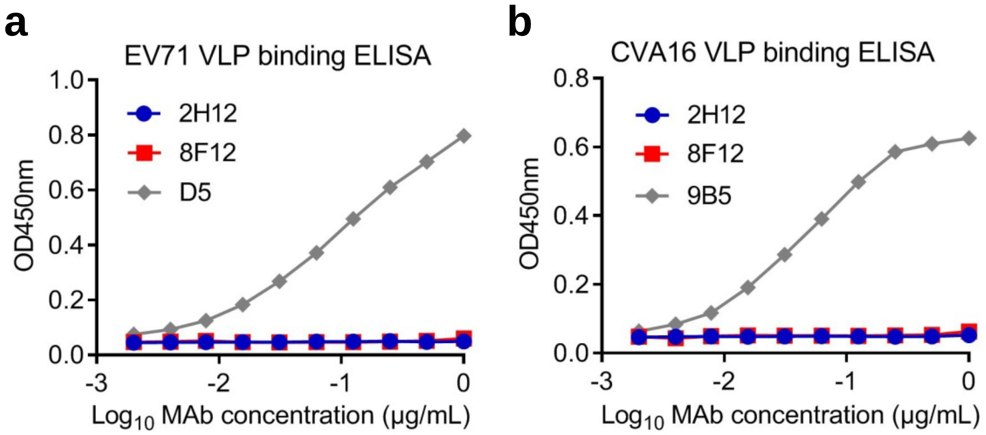

Supplementary Figure 2. Reactivities of anti-EV-D68 MAbs (2H12 and 8F12) towards EV71 VLP **(a)**, and CVA16 VLP **(b)** determined by ELISA. Anti-EV71 MAb D5 and anti-CVA16 MAb 9B5 were used as positive controls for detection of EV71 VLP and CVA16 VLP, respectively. Data are expressed as mean ± SD of triplicate wells.

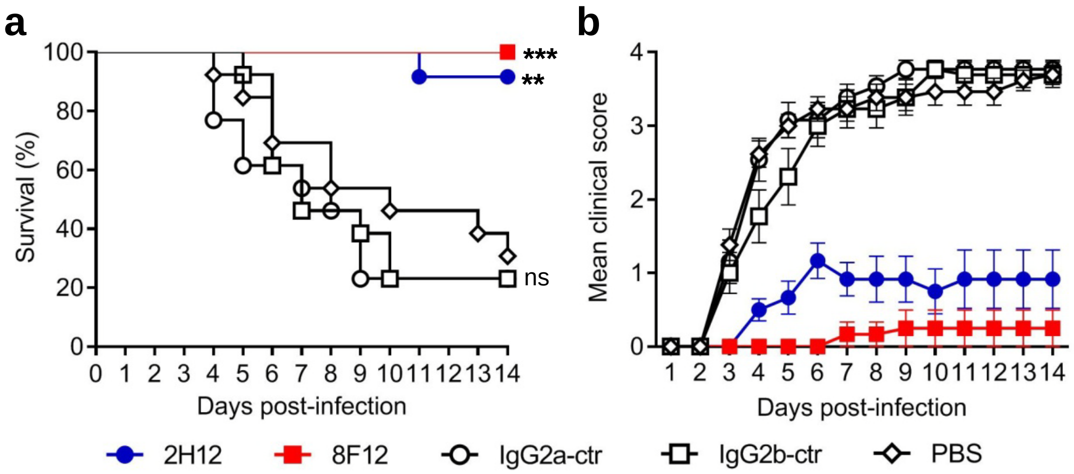

Supplementary Figure 3. Prophylactic efficacy of MAbs 2H12 and 8F12 against EV-D68 infection in neonatal mice. Groups of ICR mice (age < 24h; n = 12–14/group) were i.p. injected with PBS, 10 µg/g of 2H12, 8F12, IgG2a isotype control (ctr) MAb (1C11), or IgG2b isotype control MAb (1F4) and one day later challenged with strain 18947. The challenged mice were monitored daily for (**a**) survival and (**b**) clinical score. Clinical scores were graded as follows: 0, healthy; 1, lethargy and reduced mobility; 2, limb weakness; 3, limb paralysis; 4, death. Survival of mice in each antibody-treated groups was compared to the PBS control group. Statistical significance was determined by Log-rank (Mantel-Cox) test and was indicated as follows: ns., no significant difference ($p \geq 0.05$); **, $p < 0.01$; ***, $p < 0.001$. In panel **a**, p value between the 8F12 group and the PBS control group is 0.0004; p value between the 2H12 group and the PBS group is 0.0017. Error bars represent SEM.

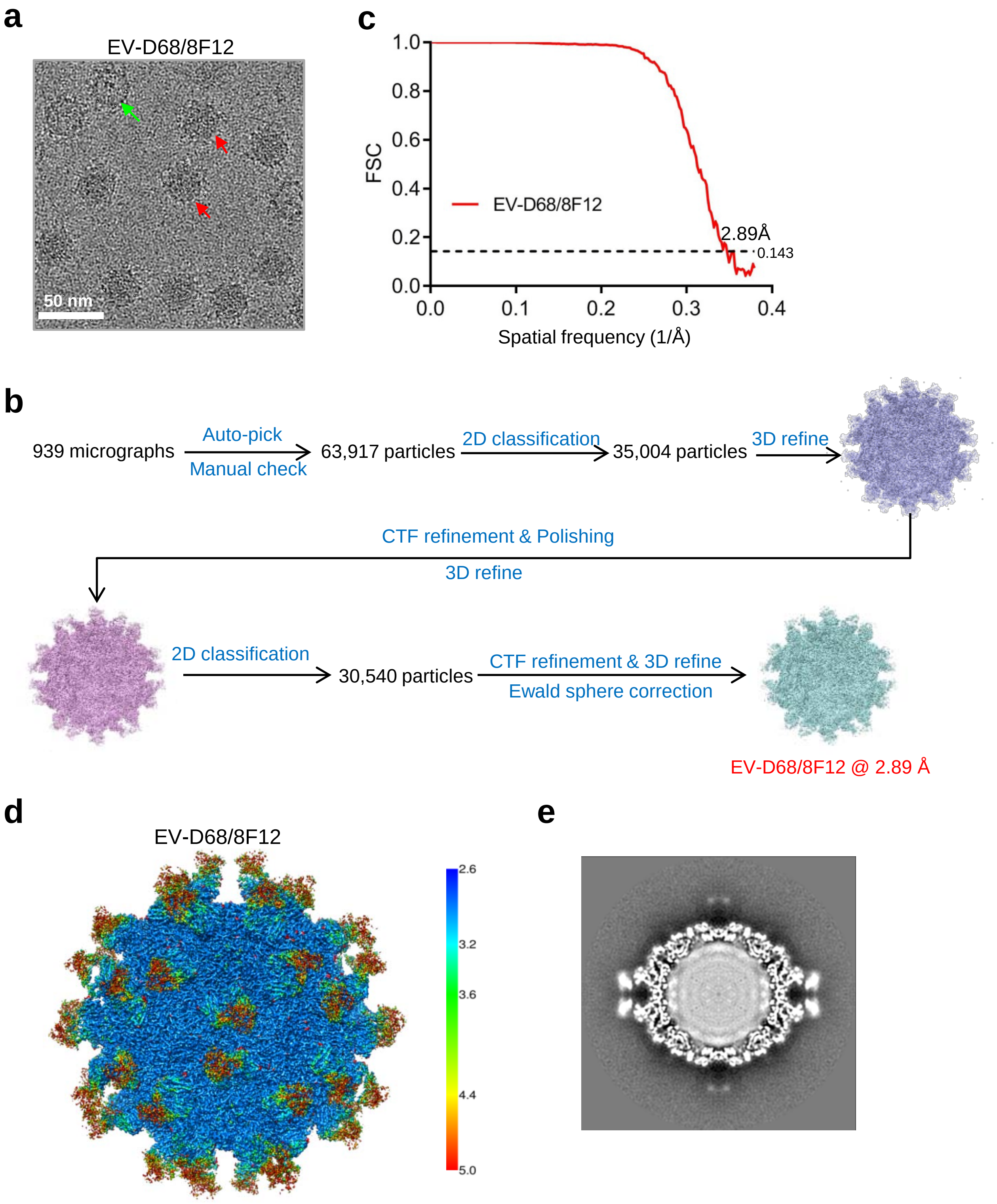

Supplementary Figure 4. Cryo-EM structural analysis of EV-D68/8F12 complex. **(a)** Cryo-EM image of the EV-D68/8F12 complex, randomly chosen from the raw images**.** Bar = 50 nm. Red and green arrows indicate full and empty particles bound by the Fabs, respectively. **(b)** Flowchart of the 3D reconstruction process for the EV-D68/8F12 complex. **(c)** Resolution assessment of the cryo-EM map of EV-D68/8F12 by Fourier shell correlation (FSC) at 0.143 criterion. **(d)** Local resolution evaluation of the cryo-EM map of EV-D68/8F12 by Relion 3.0. The resolution color bar (in Å) is also shown. **(e)** Central section of the EV-D68/8F12 cryo-EM map.

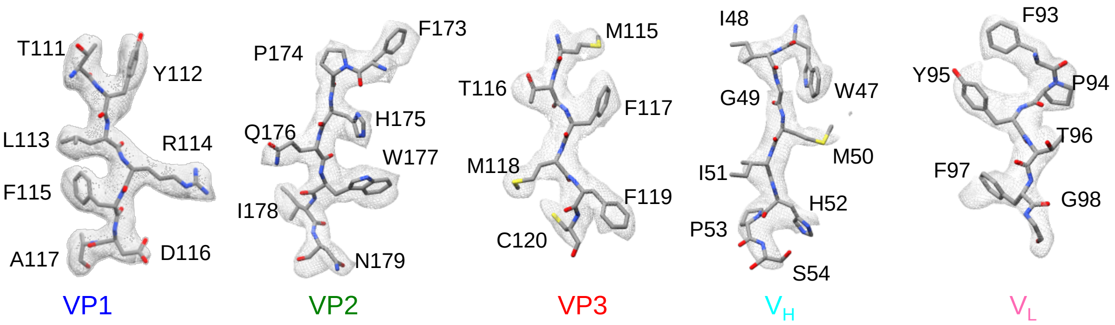

Supplementary Figure 5. Representative high-resolution structural features of the EV-D68/8F12 complex. The density map (gray mesh) matches the corresponding atomic model (in sticks) very well.

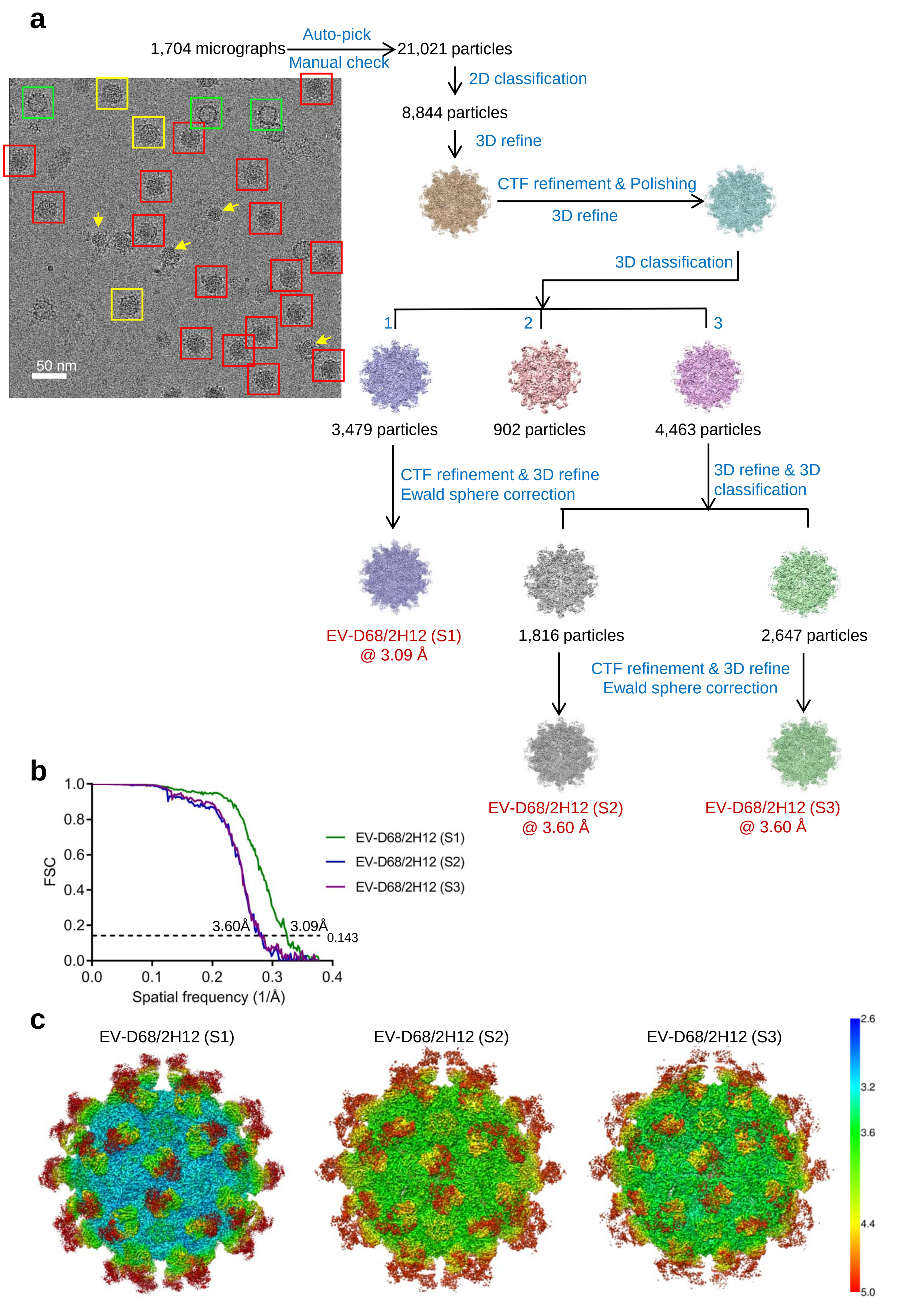
a
1,704 micrographs
Auto-pick
Manual check
21,021 particles
2D classification
8,844 particles
3D refine
CTF refinement & Polishing
3D refine
3D classification
1
2
3
3,479 particles
902 particles
4,463 particles
CTF refinement & 3D refine
Ewald sphere correction
3D refine & 3D classification
50 nm
EV-D68/2H12 (S1)
@ 3.09 Å
1,816 particles
2,647 particles
CTF refinement & 3D refine
Ewald sphere correction
EV-D68/2H12 (S2)
@ 3.60 Å
EV-D68/2H12 (S3)
@ 3.60 Å
b
FSC
1.0
0.8
0.6
0.4
0.2
0.0
0.0
0.1
0.2
0.3
0.4
Spatial frequency (1/Å)
3.60Å
3.09Å
0.143
EV-D68/2H12 (S1)
EV-D68/2H12 (S2)
EV-D68/2H12 (S3)
c
EV-D68/2H12 (S1)
EV-D68/2H12 (S2)
EV-D68/2H12 (S3)
2.6
3.2
3.6
4.4
5.0

Supplementary Figure 6. Cryo-EM structural analysis of EV-D68/2H12 complex. **(a)** Representative cryo-EM image of the EV-D68/2H12 complex and the flowchart of the 3D reconstruction process for the complex. Full particle, partially solid-core particle, and empty particle are marked in red, yellow, and green boxes, respectively. Yellow arrow indicates the broken particle induced by 2H12 binding and the exposed viral genome. **(b)** Resolution assessment of the cryo-EM reconstructions of EV-D68/2H12 by FSC at 0.143 criterion. **(c)** Local resolution evaluation of cryo-EM maps of EV-D68/2H12 (states S1, S2, and S3) by Relion 3.0. The resolution color bar (in Å) is shown.

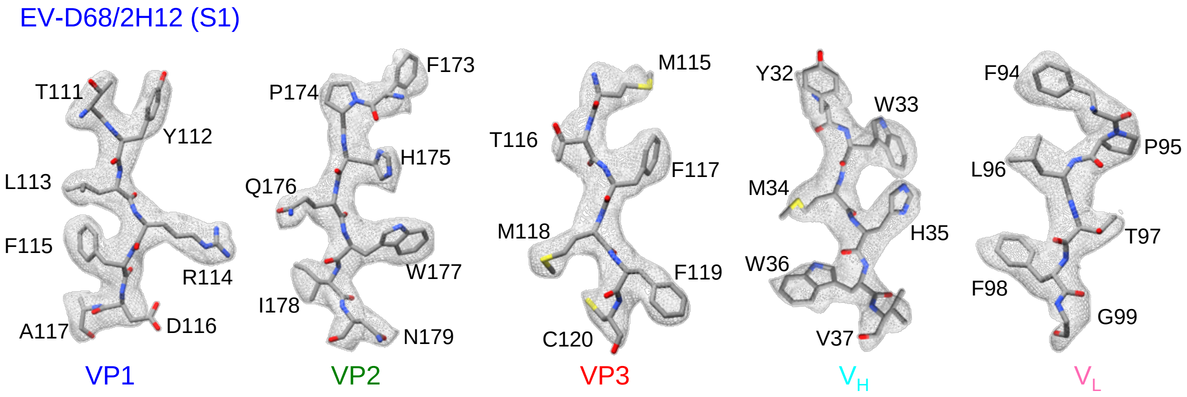

Supplementary Figure 7. Representative high-resolution structural features of the of the EV-D68/2H12 (S1) map. The density maps (gray mesh) matches the corresponding atomic model (in sticks) very well.

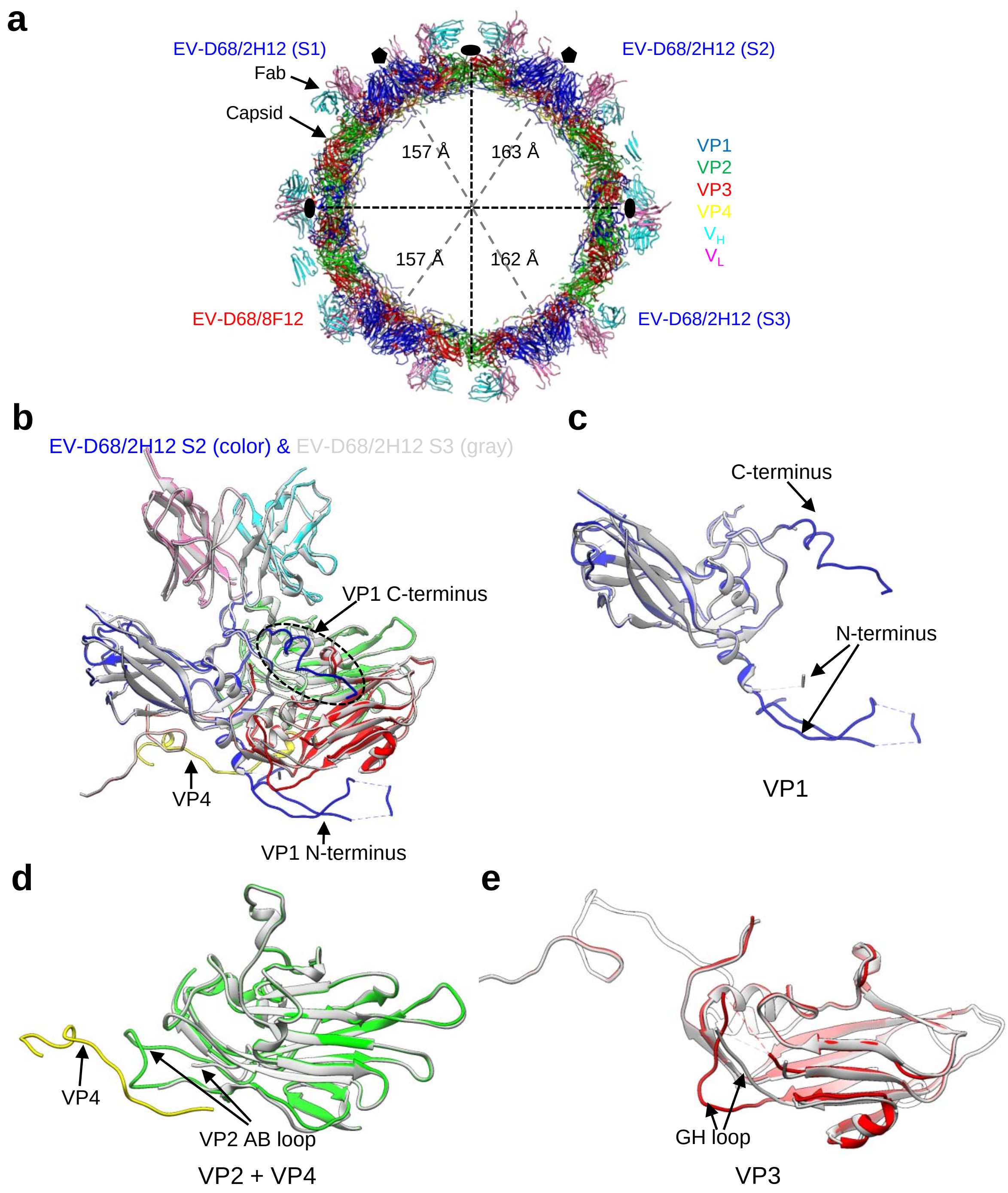

Supplementary Figure 8. Structural comparison of the EV-D68/8F12 and EV-D68/2H12 (states S1, S2, and S3), and comparison between the S2 and S3 states of EV-D68/2H12. **(a)** Central slabs (ribbon) of the EV-D68/8F12 and EV-D68/2H12 (S1, S2, and S3) structures. Only one quarter of each structure is shown, to demonstrate the capsid expansion of EV-D68/2H12 S2 (top right) and S3 (bottom right) with respect to EV-D68/8F12 (bottom left) and EV-D68/2H12 S1 (top left). Radius of capsid shell for each structure is shown. For radius measurement, we first measured the diameter of the virus capsid through measuring the distance between the CG2 atom in residue V231 of VP1 from protomers located in the opposite direction on the diagonal, then divided the diameter by two to deduce the radius value. This atom is located at the most protruding part of the virus capsid. Black oval and pentagon indicate locations of the two-fold and five-fold axes, respectively. **(b-e)** Structural comparison between the EV-D68/2H12 S2 (color) and S3 (gray) states for the protomer **(b)** and individual subunit **(c-e)**. The major conformational differences between the two states are indicated by arrows.

a

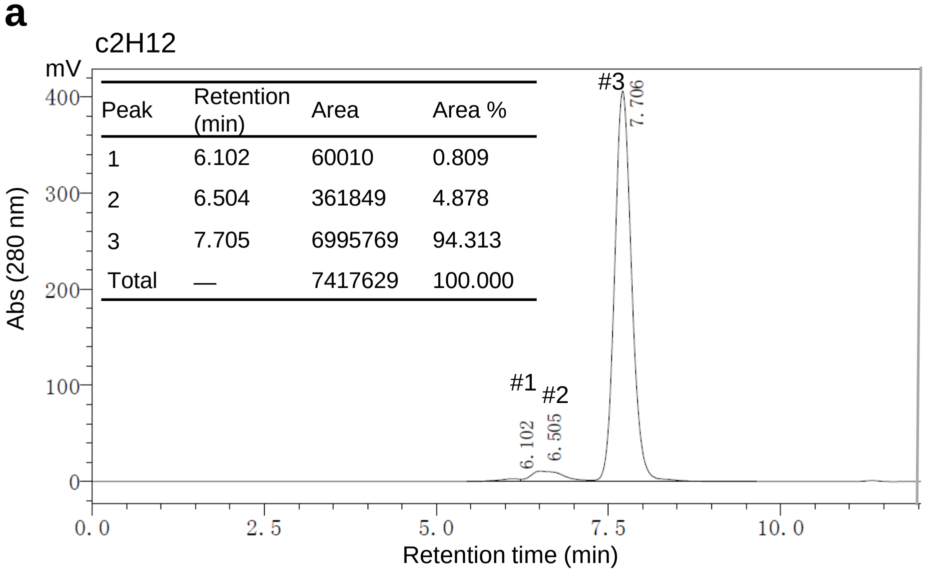

Peak	Retention (min)	Area	Area %
1	6.102	60010	0.809
2	6.504	361849	4.878
3	7.705	6995769	94.313
Total	—	7417629	100.000

b

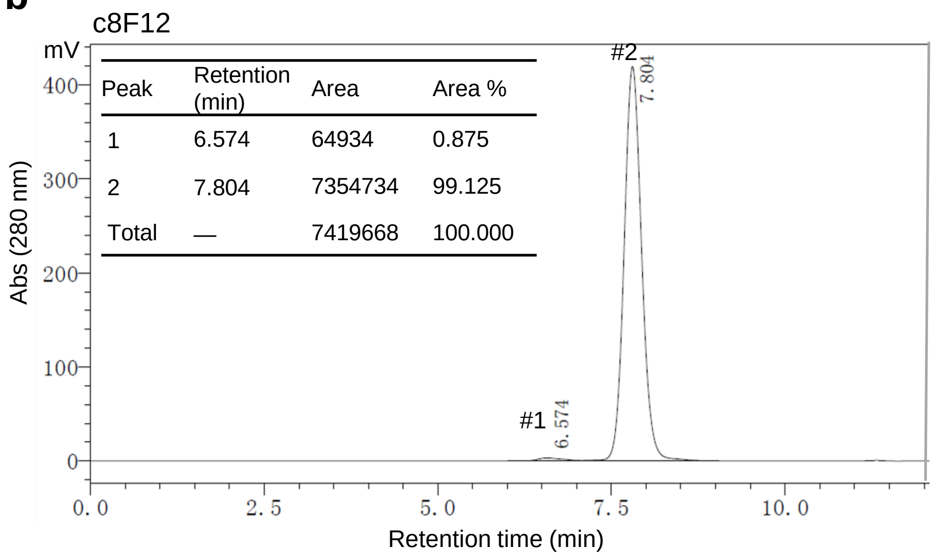

Peak	Retention (min)	Area	Area %
1	6.574	64934	0.875
2	7.804	7354734	99.125
Total	—	7419668	100.000

Supplementary Figure 9. The purity of the recombinant MAbs c2H12 **(a)** and c8F12 **(b)** was determined by size exclusion chromatography. The retention time and area of all the elution peaks are shown in the inset. The third elution peak of c2H12 and the second peak of c8F12 represent antibody monomer, whereas the first and second peaks of c2H12 and the first peak of c8F12 account for antibody aggregate and/or dimer.

Supplementary Table 1. A summary of all the EV-D68 strains used in this study.

EV-D68 strains	Abbreviation	Clade	Source (catalog)	Genbank ID
US/MO/14-18947	18947	B	ATCC (VR-1823)	KM851225
US/MO/14-18950 [a]	18950	B	P1 gene was synthesized	KM851228
US/KY/14-18953	18953	D	ATCC (VR-1825)	KM851231
Fermon	Fermon	Prototype	ATCC (VR-1826)	AY426531

[a] VLP vaccine strain.

Supplementary Table 2. Cryo-EM data collection and refinement statistics.

	**EV-D68/8F12**	**EV-D68/2H12 (S1)**	**EV-D68/2H12 (S2)**	**EV-D68/2H12 (S3)**
**Data collection**				
EM equipment	Titan Krios		Titan Krios	
Voltage(kV)	300		300	
Detector	K2 Summit		K2 Summit	
Pixel size (Å)	1.318		1.318	
Electron dose ($e^{-}/Å^{2}$)	38		38	
Exposure time (s)	7.6		7.6	
Frames	38		38	
Defocus range (μm)	-0.5 to -1.8		-0.4 to -1.6	
**Reconstruction**				
Software	Relion		Relion	
Raw micrographs	939		1,704	
Final particles	30,540	3,479	1,816	2,647
Final resolution (Å)	2.89	3.09	3.60	3.60
**Atomic modeling**				
Software	Phenix & COOT	Phenix & COOT	Phenix & COOT	Phenix & COOT
Ramachandran favored	96.10%	95.84%	94.84%	93.22%
Ramachandran allowed	3.90%	4.06%	5.16%	6.55%
Ramachandran outliers	0.00%	0.10%	0.00%	0.23%

Supplementary Table 3. RMSD values between capsid protomers of different states of EV-D68.

RMSD (Å)	EV-D68/8F12	EV-D68/2H12 (S1)	EV-D68/2H12 (S2)	EV-D68/2H12 (S3)
Mature EV-D68 (PDB: 6CSG)	0.53	0.86	1.99	2.51
EV-D68 A-particle (PDB: 6CS6)	4.09	4.20	3.52	1.61
EV-D68 E1 particle (PDB: 6CS3)			1.21	1.86

Supplementary Table 4. Notable interaction contacts between mature EV-D68 virion and 8F12.

EV-D68		Distance (Å)	8F12		Interaction
Residue	Location		Residue	Location	
R1085 [a] [NH2]	VP1 BC loop	3.87	S62 [OG]	LC-FR3 [b]	H-bond
N1206 [ND2]	VP1 GH loop	3.11	S26 [O]	LC-FR1	H-bond
N2136 [O]	VP2 EF loop	3.01	W33 [NE1]	HC-CDR1 [c]	H-bond
T2139 [OG1]	VP2 EF loop	2.95	W90 [O]	LC-CDR3	H-bond
T2139 [OG1]	VP2 EF loop	3.13	Y95 [OH]	LC-CDR3	H-bond
S2140 [N]	VP2 EF loop	2.83	S91 [O]	LC-CDR3	H-bond
S2140 [O]	VP2 EF loop	3.68	F93 [N]	LC-CDR3	H-bond
A3244 [N]	VP3 C-terminus	3.07	Y31 [OH]	LC-CDR1	H-bond

[a] The N-terminal amino acids of VP1, VP2, and VP3 are 1001, 2001, and 3001, respectively.

[b] LC, light chain.

[c] HC, heavy chain.

Supplementary Table 5. Notable interaction contacts between mature EV-D68 virion and 2H12 (S1).

EV-D68		Distance (Å)	2H12		Interaction
Residue	Location		Residue	Location	
K1205 [O]	VP1 GH loop	2.81	D1 [N]	LC-FR1	H-bond
N1206 [O]	VP1 GH loop	3.42	Q27 [NE2]	LC-CDR1	H-bond
H2135 [O]	VP2 EF loop	2.78	W33 [NE1]	HC-CDR1	H-bond
H2135 [ND1]	VP2 EF loop	3.62	D52 [OD2]	HC-CDR2	Salt bridge/H-bond
N2136 [ND2]	VP2 EF loop	2.65	D31 [O]	HC-CDR1	H-bond
N2136 [O]	VP2 EF loop	3.25	G101 [N]	HC-CDR3	H-bond
N2138 [ND2]	VP2 EF loop	2.55	G101 [O]	HC-CDR3	H-bond
T2139 [OG1]	VP2 EF loop	2.73	Y91 [O]	LC-CDR3	H-bond
S2140 [N]	VP2 EF loop	3.06	A92 [O]	LC-CDR3	H-bond
S2140 [O]	VP2 EF loop	3.72	S93 [OG]	LC-CDR3	H-bond
S2140 [O]	VP2 EF loop	3.28	F94 [N]	LC-CDR3	H-bond
D2144 [OD2]	VP2 EF loop	2.64	K65 [NZ]	HC-FR3	Salt bridge/H-bond
D2145 [OD2]	VP2 EF loop	3.20	T59 [OG1]	HC-FR3	H-bond
H2157 [NE2]	VP2 EF loop	3.88	S54 [OG]	HC-CDR2	H-bond
H3240 [NE2]	VP3 C-terminus	2.63	S32 [OG]	LC-CDR1	H-bond
A3244 [O]	VP3 C-terminus	2.78	R66 [NH2]	LC-FR3	H-bond
Q3247 [NE2]	VP3 C-terminus	3.88	R66 [O]	LC-FR3	H-bond

Supplementary Table 6. Surface area of EV-D68 buried by MAb 8F12 and 2H12 (in state S1) determined by the PISA server.

MAb	Interface area (Å^2)	
	total	Contribution
8F12-V_H	343.4	30.4%
8F12-V_L	786.7	69.6%
8F12	1130.1	100.0%
2H12-V_H	511.1	44.2%
2H12-V_L	644.8	55.8%
2H12	1155.9	100.0%

Supplementary Table 7. A complete list of all primers used in this study

Plasmid or gene	Primer name	Primer sequence
pcDNA3.4-8F12-Fd-his	8F12-VH-F	GTCCTCCTGACTGGGGTGAGGGCCCAGGTCCAACTGCAGCAGCCTGGG
	8F12-VH-R	GACTGATGGGGGTGTTGTTTTAGCTGCAGAGACAGTGACCAGAGTC
pcDNA3.4-8F12-Kappa	8F12-Vκ-F	GTCCTCCTGACTGGGGTGAGGGCCCAAATTGTTCTCACCCAGTCTCCA
	8F12-Vκ-R	GATACAGTTGGTGCAGCATCTGCTCTTTTTATTTCCAGCTTGGTCC
pcDNA3.4-c2H12-hIgG1	c2H12-VH-F	TCCTGACTGGGGTGAGGGCCCAGGTCCAACTGCAGCAGCCTGGGGC
	c2H12-VH-R	GATGGGCCCTTGGTGCTAGCTGCAGAGACAGTGACCAGAGTCCCTT
pcDNA3.4-c2H12-hκ	c2H12-Vκ-F	GTCCTCCTGACTGGGGTGAGGGCCGACATCCAGATGACCCAGTCTCCA
	c2H12-Vκ-R	GACAGATGGTGCAGCCACCGTACGTTTCAGCTCCAGCTTGGTCCCAGC
pcDNA3.4-c8F12-hIgG1	c8F12-VH-F	GTCCTCCTGACTGGGGTGAGGGCCCAGGTCCAACTGCAGCAGCCTGGG
	c8F12-VH-R	GACCGATGGGCCCTTGGTGCTAGCTGCAGAGACAGTGACCAGAGTCCC
pcDNA3.4-c8F12-hκ	c8F12-Vk-F	GTCCTCCTGACTGGGGTGAGGGCCCAAATTGTTCTCACCCAGTCTCCA
	c8F12-Vk-R	GACAGATGGTGCAGCCACCGTACGTTTTATTTCCAGCTTGGTCCCCCC
VP1 gene of strain 18947 (QPCR)	18947-VP1-F	CGAGAGCATCATCAAAACAGCGACC
	18947-VP1-R	CACTGTGCGAGTTTGTATGGCTTCT
β-actin gene (QPCR)	β-actin-F	GGACTTCGAGCAAGAGATGG
	β-actin-R	AGCACTGTGTTGGCGTACAG
VP1 gene of strain 18953 (QPCR)	18953-VP1-F	GGAAGCCATACAAACTCG
	18953-VP1-R	TTCGTGCTTCAGATGAGGTG
