# Supplementary material for: Functional and structural characterization of a two-MAb cocktail for delayed treatment of enterovirus D68 infections
Source: Nat Commun. 2021 May 18;12:2904. doi: 10.1038/s41467-021-23199-5 (PMC8131599; doi:10.1038/s41467-021-23199-5)
Supplement: Supplementary file 3 — Reporting Summary [file 41467_2021_23199_MOESM3_ESM.pdf]

nature research

Corresponding author(s): Zhong Huang

Last updated by author(s): Mar 31, 2021

Reporting Summary

Nature Research wishes to improve the reproducibility of the work that we publish. This form provides structure for consistency and transparency in reporting. For further information on Nature Research policies, see our Editorial Policies and the Editorial Policy Checklist.

Statistics

For all statistical analyses, confirm that the following items are present in the figure legend, table legend, main text, or Methods section.

n/a	Confirmed	
☐	☒	The exact sample size (*n*) for each experimental group/condition, given as a discrete number and unit of measurement
☐	☒	A statement on whether measurements were taken from distinct samples or whether the same sample was measured repeatedly
☐	☒	The statistical test(s) used AND whether they are one- or two-sided<br>*Only common tests should be described solely by name; describe more complex techniques in the Methods section.*
☒	☐	A description of all covariates tested
☒	☐	A description of any assumptions or corrections, such as tests of normality and adjustment for multiple comparisons
☐	☒	A full description of the statistical parameters including central tendency (e.g. means) or other basic estimates (e.g. regression coefficient) AND variation (e.g. standard deviation) or associated estimates of uncertainty (e.g. confidence intervals)
☐	☒	For null hypothesis testing, the test statistic (e.g. *F*, *t*, *r*) with confidence intervals, effect sizes, degrees of freedom and *P* value noted<br>*Give P values as exact values whenever suitable.*
☒	☐	For Bayesian analysis, information on the choice of priors and Markov chain Monte Carlo settings
☒	☐	For hierarchical and complex designs, identification of the appropriate level for tests and full reporting of outcomes
☒	☐	Estimates of effect sizes (e.g. Cohen's *d*, Pearson's *r*), indicating how they were calculated

Our web collection on statistics for biologists contains articles on many of the points above.

Software and code

Policy information about availability of computer code

Data collection	FEI TEM user interface 2.15.3, SerialEM 3.7.0
Data analysis	GraphPad Prism version 8, MotionCor2, RELION 3.0, CTFFIND 4.1.8, Gctf v0.50, Phenix 1.10.1, COOT 0.8.3, Molprobilty 1.10.1-2155, UCSF Chimera 1.10.2, PDBePISA 1.48, RIVEM 4.3, Octet Data Analysis 11.0, IgBLAST tool 1.14.0.

For manuscripts utilizing custom algorithms or software that are central to the research but not yet described in published literature, software must be made available to editors and reviewers. We strongly encourage code deposition in a community repository (e.g. GitHub). See the Nature Research guidelines for submitting code & software for further information.

Data

Policy information about availability of data

All manuscripts must include a data availability statement. This statement should provide the following information, where applicable:

- Accession codes, unique identifiers, or web links for publicly available datasets
- A list of figures that have associated raw data
- A description of any restrictions on data availability

The authors declare that the data supporting the findings are available from the corresponding authors upon reasonable request. Cryo-EM map determined in the EV-D68/8F12 dataset has been deposited at the Electron Microscopy Data Bank with accession code of EMD-31056, and associated atomic model have been deposited in the Protein Data Bank with accession code of 7EC5. Cryo-EM maps (S1, S2, and S3) determined in the EV-D68/2H12 dataset have been deposited at the Electron Microscopy Data Bank with accession codes of EMD-31055, EMD-31054, and EMD-31060, respectively, and related models have been deposited in the Protein Data Bank under accession codes of 7EBZ, 7EBR, and 7ECY, respectively. The sequences of 2H12-VH, 2H12-VL, 8F12-VH, and 8F12-VL have been deposited in GenBank with the accession codes MW627209, MW627210, MW627211, and MW627212, respectively. Source data are provided with this paper.

Field-specific reporting

Please select the one below that is the best fit for your research. If you are not sure, read the appropriate sections before making your selection.

☒ Life sciences ☐ Behavioural & social sciences ☐ Ecological, evolutionary & environmental sciences

For a reference copy of the document with all sections, see nature.com/documents/nr-reporting-summary-flat.pdf

Life sciences study design

All studies must disclose on these points even when the disclosure is negative.

Sample size	Trial experiments done previously were used to determine sample size with adequate statistical power. For animal experiments, each group included 12–27 mice. The sample size was sufficient for a good statistical analysis.
Data exclusions	No data were excluded from the analysis.
Replication	Experimental findings were reliably reproduced. Most of the experiments (including ELISA binding assay, neutralization assay, real-time PCR, and hemagglutination inhibition assay) were replicated two or three times.
Randomization	Animals were randomly divided into experimental groups. Randomizations are irrlevant to in vitro cell line based assays or biochemical assays.
Blinding	The investigators were not completely blinded during the experiment.

Reporting for specific materials, systems and methods

We require information from authors about some types of materials, experimental systems and methods used in many studies. Here, indicate whether each material, system or method listed is relevant to your study. If you are not sure if a list item applies to your research, read the appropriate section before selecting a response.

Materials & experimental systems

n/a	Involved in the study
☐	☒ Antibodies
☐	☒ Eukaryotic cell lines
☒	☐ Palaeontology and archaeology
☐	☒ Animals and other organisms
☒	☐ Human research participants
☒	☐ Clinical data
☒	☐ Dual use research of concern

Methods

n/a	Involved in the study
☒	☐ ChIP-seq
☒	☐ Flow cytometry
☒	☐ MRI-based neuroimaging

Antibodies

Antibodies used	Anti-Mouse IgG (Fc specific)–Peroxidase antibody produced in goat (Sigma, A0168-1ML); Rabbit Anti-Human IgG H&L (HRP) (abcam, ab6759); anti-EV71 MAb D5, anti-CVA16 MAb 9B5, anti-ZIKV MAb 1C11 and anti-HCV MAb 1F4 were prepared in our lab.
Validation	The specifications of commercially available antibodies can be found on the manufacture's website using their catalogue numbers. The specificity of anti-EV71 MAb D5 and anti-CVA16 MAb 9B5 were demonstrated in the present study and have also been demonstrated previously (Ku et al, 2012. J Virol Methods 186, 193-197; Ku Z, et al, 2014. Vaccine 32, 4296-4303). Anti-ZIKV MAb 1C11 has been validated previously (Qu et al, 2020. Cell Discovery 6:5). Anti-HCV MAb 1F4 was used as isotype control (its isotype was determined by ELISA using the SBA Clonotyping System-HRP kit).

Eukaryotic cell lines

Policy information about cell lines

Cell line source(s)	Human rhabdomyosarcoma (RD) cells, ATCC, CCL-136; SP2/0 myeloma cells, the Cell Bank of the Chinese Academy of Sciences (Shanghai, China); HEK 293F suspension cells, thermo fisher.
Authentication	The cell lines were not authenticated further after purchase.
Mycoplasma contamination	Cell lines have not recently been tested for mycoplasma contamination
Commonly misidentified lines (See ICLAC register)	No commonly misidentified lines were used

Animals and other organisms

Policy information about studies involving animals; ARRIVE guidelines recommended for reporting animal research

Laboratory animals	BALB/c mice (female; 6–8 week old) and ICR mice (female; pregnant) . All mice were purchased from Shanghai Laboratory Animal Center (SLAC, China).
Wild animals	No wild animals were used.
Field-collected samples	No
Ethics oversight	The animal studies were approved by the Institutional Animal Care and Use Committee at the Institut Pasteur of Shanghai.

Note that full information on the approval of the study protocol must also be provided in the manuscript.
